# Fabrication and characterization of Agarwood extract-loaded nanocapsules and evaluation of their toxicity and anti-inflammatory activity on RAW 264.7 cells and in zebrafish embryos

**DOI:** 10.1080/10717544.2021.2012307

**Published:** 2021-12-13

**Authors:** Manar A. Eissa, Yumi Z. H.-Y. Hashim, Mohd Hamzah Mohd Nasir, Yusilawati Ahmad Nor, Hamzah Mohd. Salleh, Muhammad Lokman Md. Isa, Saripah S. S. Abd-Azziz, Nor Malia Abd Warif, Eman Ramadan, Noha M. Badawi

**Affiliations:** a International Institute for Halal Research and Training (INHART), International Islamic University Malaysia (IIUM), Kuala Lumpur, Malaysia; b Center for Drug Research and Development (CDRD), The British University in Egypt (BUE), Cairo, Egypt; c Department of Biotechnology, Kulliyyah of Science, International Islamic University Malaysia (IIUM), Kuantan, Pahang, Malaysia; d Central Research and Animal Facility (CREAM), Kulliyyah of Science, International Islamic University Malaysia (IIUM), Kuantan, Pahang, Malaysia; e Department of Biotechnology Engineering, Kulliyyah of Engineering, International Islamic University Malaysia (IIUM), Kuala Lumpur, Malaysia; f Kulliyah of Nursing, International Islamic University Malaysia (IIUM), Jalan Sultan Ahmad Shah, Kuantan, Pahang, Malaysia; g Faculty of Science and Mathematics, Sultan Idris Education University, Perak, Tanjung Malim, Malaysia; h Biomedical Sciences Program, Faculty of Health Sciences, Universiti Kebangsaan Malaysia, Kuala Lumpur, Malaysia; i Department of Pharmacology and Toxicology, Faculty of Pharmacy, The British University in Egypt (BUE), Cairo, Egypt; j Department of Pharmaceutics and Pharmaceutical Technology, Faculty of Pharmacy, The British University in Egypt (BUE), Cairo, Egypt

**Keywords:** *Aquilaria malaccensis*, polymeric nanocapsules, anti-inflammatory, RAW 264.7, zebrafish

## Abstract

*Aquilaria malaccensis* has been traditionally used to treat several medical disorders including inflammation. However, the traditional claims of this plant as an anti-inflammatory agent has not been substantially evaluated using modern scientific techniques. The main objective of this study was to evaluate the anti-inflammatory effect of *Aquilaria malacensis* leaf extract (ALEX-M) and potentiate its activity through nano-encapsulation. The extract-loaded nanocapsules were fabricated using water-in-oil-in-water (*w/o/w*) emulsion method and characterized via multiple techniques including DLS, TEM, FTIR, and TGA. The toxicity and the anti-inflammatory activity of ALEX-M and the extract-loaded nanocapsules (ALEX-M-PNCs) were evaluated in-vitro on RAW 264.7 macrophages and *in-vivo* on zebrafish embryos. The nanocapsules demonstrated spherical shape with mean particle diameter of 167.13 ± 1.24 nm, narrow size distribution (PDI = 0.29 ± 0.01), and high encapsulation efficiency (87.36 ± 1.81%). ALEX-M demonstrated high viability at high concentrations in RAW 264.7 cells and zebrafish embryos, however, ALEX-M-PNCs showed relatively higher cytotoxicity. Both free and nanoencapsulated extract expressed anti-inflammatory effects through significant reduction of the pro-inflammatory mediator nitric oxide (NO) production in LPS/IFNγ-stimulated RAW 264.7 macrophages and zebrafish embryos in a concentration-dependent manner. The findings highlight that ALEX-M can be recognized as a potential anti-inflammatory agent, and its anti-inflammatory activity can be potentiated by nano-encapsulation. Further studies are warranted toward investigation of the mechanistic and immunomodulatory roles of ALEX-M.

## Introduction

Inflammation is a complex pathophysiological process that plays a vital role in protecting the body against internal or external noxious stimuli such as pathogens, injuries, chemicals and radiation, in order to restore body performance and tissue homeostasis (Izzany et al., 2018; Pesic and Greten, 2016; Ruslan Medzhitov, 2008). However, it was found that chronic unresolved inflammation is an underlying cause in many degenerative diseases and major malignancies in human beings ranging from minor to serious conditions such as diabetes, atherosclerosis, rheumatoid arthritis, cancer and cardiovascular diseases (Fürst and Zündorf, 2014; Medzhitov, 2008). The most common drugs for treatment of inflammatory disorders are non-steroidal anti-inflammatory drugs (NSAIDs) and corticosteroids (Gautam and Jachak, 2009). Nevertheless, high doses and prolonged use of synthetic anti-inflammatory medications may lead to intolerable side effects. For instance, NSAIDS were reported to cause gastrointestinal bleeding, hepatic complications and cardiovascular problems (Izzany et al., 2018), while the prolonged use of corticosteroids leads to increased blood glucose level, hypertension, skin disorders and gastrointestinal upsets (Gautam and Jachak, 2009; Schacke et al., 2002). Accordingly, the discovery of effective and safe natural alternatives for the treatment of inflammation has attracted the attention of researchers, aiming to obviate the adverse effects of synthetic medications.

Currently, almost 80% of world populations depend on traditional herbal medicine (Tiwari, 2008), especially in less developed countries where herbal medicine is most affordable (Fürst and Zündorf, 2014). Across communities all over the world, traditional medicines administered in the form of herbal drinks have been known to relieve inflammation. Malaysia is among the 12 megadiversity countries in the world where around 2000 plant species are reported to exhibit health benefit properties (Izzany et al., 2018). A number of Malaysian plants were reported to exhibit anti-inflammatory activity as reported by multiple researchers (Abas et al., 2006; Chung et al., 2009; Karimi et al., 2013; Oskoueian et al., 2011). One of the local Malaysian plants, *Aquilaria malaccensis* is a large and fast growing tropical evergreen tree that belongs to family Thymelaeaceae and is native to Indomalesian rainforests. The tree is also distributed in other countries such as Myanmar, Singapore and Thailand (UNEP WCMC, 2014). Plant materials from this tree have been used traditionally to alleviate multiple diseases such as rheumatism, ulcer, skin diseases, pain and others (Chakrabarty et al., 1994). In addition, herbal tea processed from *Aquilaria* leaves has gained wide popularity in Asian countries to relief morbid conditions including inflammatory-related disorders (Adam et al., 2017). Yet, the anti-inflammatory effect of this species has not been fully investigated.

Although the use of natural medicinal herbs is highly recommended due to their safety and good therapeutic activity, the complexity of their biologically-active constituents renders the conventional drug delivery forms deficient in achieving the target outcome (Armendáriz-barragán et al., 2016). The conventional means of drug delivery are usually associated with certain limitations such as poor bioavailability, lack of selectivity, low activity and unfavorable toxicity. However, the emergence of nano-sized drug delivery systems offered some promising solutions with their exceptional characteristics in increasing drug bioavailability, improving drug-targeting, lowering drug dosing, enhancing efficacy and minimizing side effects (Cismaru and Popa, 2010; Hosseini et al., 2016). Polymeric nanoparticles in particular are considered amongst the most efficient drug delivery systems and they gained significant attention in numerous researches and applications in the past few years due to their safety, flexibility and their ability to deliver drugs to targeted organs in a controlled drug release manner (Fornaguera and Solans, 2018; Liechty et al., 2010). The advantages of using polymeric nanocapsules in delivering herbal extracts include the enhancement of their biological activity and ability to overcome the challenges related to their solubility, bioavailability, stability and release (Bennet et al., 2014; Kim et al., 2016; Kwon et al. 2012; Mendes et al. 2017; Ngadiwiyana et al. 2017; Strasser et al., 2014; Suman and Gupta, 2013).

The use of natural polymers for drug encapsulation has distinctive edge over synthetic polymers due to their biocompatibility, biodegradability, abundance (since they can be derived from renewable resources) and reasonable cost of production (Katmıs et al., 2018; Nazarzadeh et al., 2019; Vishakha et al., 2017). In the present study, Tragacanth gum (TG) was selected to be used as a coating material for encapsulation of the extract. TG is a highly branched natural anionic polysaccharide that is obtained from the branches of different species of *Astragalus* plant as dried exudate (Mohammadifar et al., 2006; Nazarzadeh et al., 2019). It is highly hydrophilic, nontoxic and biocompatible which makes it a good candidate to be used in different fields (Kiani et al., 2012). The hydroxyl and carboxylic acid groups are the active sites for cross-linking and interaction with other groups, henceforth, TG can be used for encapsulation of plant extracts and volatile oils using cross linking technique as demonstrated in several studies (Ghayempour et al., 2015a, 2016a; Ghayempour and Montazer, 2017; Hosseini et al., 2016).

While LPS-induced NO production in RAW 264.7 cells has been broadly used as cell model to study anti-inflammatory extracts in-vitro (Gasparrini et al., 2017; Jo 2018; Lee et al., 2012; Won et al., 2018), zebrafish embryo recently gained acceptance as a promising model for screening pharmacological potential and toxicity of phytochemicals. Recent work documented various advantages of using embryonic zebrafish model over in-vitro cell culture assays and other in vivo mammalian models. This include permeability of the chorion which allows drug diffusion into the embryo, and the optical transparency of the embryo allowing noninvasive and direct observation of the body organs (Evensen et al., 2016; Syahbirin et al., 2017). In addition, the genome of zebrafish and humans share high similarity with around 75% homologues (Chakraborty et al., 2016; Santoriello and Zon, 2012). Zebrafish embryo is also accepted as a proper model for assessing the anti-inflammatory effect of drugs because zebrafish has innate and acquired immune systems similar to mammals (Hwang et al., 2016; S. Lee et al., 2013). Also, the zebrafish embryos can develop immune cells similar to their mammalian equivalents (Liao et al., 2011), which makes the inflammatory pathophysiological features of zebrafish similar to human (Yang et al., 2014).

The objective of this study is to fabricate and characterize polymeric nanocapsules loaded with *Aquilaria malaccensis* leaf extract (ALEX-M). The toxicity and the anti-inflammatory activity of ALEX-M, the prepared extract-loaded nanocapsules (ALEX-M-PNCs) and the blank nanocapsules (B-PNCs) were evaluated in-vitro on RAW 264.7 macrophages and in-vivo using zebrafish embryos.

## Materials and methods

### Materials

*Aquilaria malaccensis* leaves were collected from a local plantation in Semenyih, Selangor, Malaysia. Tragacanth gum (Sigma Aldrich), AlCl_3_ (Sigma Aldrich), almond oil (Merck), Dulbecco's Modified Eagle's Medium (DMEM) high glucose media (41965039, Gibco, life technologies, MA, USA), Phenol-free DMEM (12-917 F, Gibco, life technologies, MA, USA), fetal bovine serum (10082139, GIBCO, USA), Penicillin/streptomycin 10,000 U/ml (15140122, Gibco, USA), Griess reagent kit (G4410, Sigma-Aldrich, St. Louis, MO, USA), MTT reagent (3-(4,5-dimethylthiazol-2-yl)-2,5-diphenyl tetrazolium bromide) (CT01-5 Sigma-Aldrich), Murine Interferon- γ (315-05) (PeproTech, NJ, USA) and dialysis cellulose membrane with molecular weight cutoff 12,000 Dalton (Sigma-Aldrich) were donated by the British University in Egypt (BUE). Triton X-100 (ACROS Organics) was purchased from Thermo Fisher Scientific (Waltham, Massachusetts, United States). Lipopolysaccharide (O111:B4 *E. coli*) was purchased from Merck (Darmstadt, Germany). The fluorescent probe, diaminofluorophore 4-amino-5-methylamino-2′,7′-difluorofluorescein diacetate DAF-FM-DA (Sigma-Aldrich), was purchased from Bita Lifescience Sdn. Bhd (Selangor, Malaysia). The murine macrophages cell lines RAW 264.7 (ATCC-TIB71, Rockville, MD, USA) were gifted by the National Research Center (NRC) (Cairo, Egypt). Zebrafish embryos were obtained from the Central Research and Animal Facility (CREAM), IIUM, Kuantan, Malaysia. All chemicals, analytical grade solvents (Merck, Darmstadt, Germany), glassware and consumables were provided by the British University in Egypt (BUE).

### Extraction

*Aquilaria malaccensis* dried and powdered leaves were macerated using 95% ethanol at room temperature and the extract was evaporated under reduced pressure using rotavapor (Heidolph) at 40 °C (Eissa et al., 2020). The yield percentage of the product was 7.63%. (wt/wt). The structure of the extract was elucidated in a previous study using phytochemical screening assays and spectroscopic techniques, namely, GC-MS and LC/Q-TOF-MS (Eissa et al., 2020). The resulting extract is referred to ALEX-M.

### Fabrication of nanocapsules

In this study, ALEX-M-PNCs were formulated by preparing water-in-oil-in-water (*w/o/w*) emulsion (Ghayempour et al., 2016a). The encapsulation process was initiated by sonicating mixture of ALEX-M (0.5 mg/ml), Almond oil (3 ml) and 0.1% Triton X-100 using SONICS Vibra-CellTM sonicator for 5 minutes at 100% amplitude. The prepared w/o emulsion was added to the solution containing 1% *w/v* TG and 0.1% *w/v* Triton X-100 and the mixture was stirred on magnetic stirrer (Daihan Scientific, MSH 20 D) at 1500 rpm for 10 min to obtain a steady white w/o/w emulsion. The cross-linker, AlCl_3_ (2% *w/v*), was added dropwise to the emulsion and stirred for 5 min followed by sonication in an ice bath. The emulsion was then centrifuged using cooling centrifuge (2-16KL, Sigma Laborzentrifugen GmbH, Osterode am Harz, Germany) at 4 °C for four hours and 12000 rpm and the nanocapsules were washed three times. Finally, the nanocapsules were left to dry at room temperature for further characterization. Deionized water was used in the process. B-PNCs were prepared in parallel without adding the extract.

### Characterization of polymeric nanocapsules

#### Measurement of particle size, polydispersity index (PDI) and zeta potential (ZP)

The average particle size, the particle size distribution (PDI) and zeta potential (ZP) of the fabricated nanocapsules were determined at 25 °C by dynamic light scattering (DLS) on a Malvern Zetasizer Nano ZS instrument (Malvern Instruments Ltd., Malvern, UK) after dilution with deionized water (Badawi et al., 2018).

#### Morphological analysis

Transmission electron microscope (TEM) (HRTEM, JEM 2100, Tokyo, Japan) equipped with a field emission gun operating at 200 kV was employed to study the morphology of the prepared nanocapsules (Badawi et al., 2018). A volume of 3 μL of the emulsion was applied on a 300-mesh copper grid coated with carbon. The samples were negatively stained using 2% uranyl acetate (*w/v*), followed by air drying at room temperature prior to microscopic analysis. Images of ALEX-M-PNCs were captured with a bottom-mounted Gatan CCD camera.

#### Encapsulation efficiency (EE) and loading capacity (LC)

The encapsulation efficiency was calculated indirectly using the method implied by Nur and Vasiljevic (2018) and S. Wang et al., (2012). Subsequent to centrifugation for 4 hours at 4 °C and 12000 rpm, the clear supernatant was collected and the absorbance was measured at *λ* = 257 nm in order to determine the amount of free extract using UV-Vis spectrophotometer (V-630, Jasco, Tokyo, Japan). The percentage of the extract entrapped was calculated according to the equation (Borges et al., 2018):

$$\text{Encapsulation Efficiency }(\mathrm{EE})\%=[\frac{\text{Total extract}-\text{Free extract}}{\text{Total extract}}]*100$$

Where the total extract represents the total amount of extract in the formulation and the free extract represents the unencapsulated extract in the supernatant.

The loading capacity (LC) is the amount of encapsulated material in nanocapsules, and was calculated using the formula (Xiao et al., 2017):

$$\text{Loading capacity }(\mathrm{LC})\%=\frac{\text{Weight of Encapsulated Extract}}{\text{Weight of nanocapsules}}\;\times \;100$$

#### In vitro extract release

The cumulative extract release from the nanocapsules was determined using the dialysis bag method as proposed by Badawi et al. (2018) with slight modifications. The dialysis bag used has a molecular weight of 12,000 Da which permits the diffusion of the free extract in the dissolution media while it retains the nanocapsules. The bags were firstly soaked for 24 hours in the dissolution medium (PBS, pH = 7.4). A volume equal to 1 ml of ALEX-M-PNCs was transferred into two dialysis bags formed by tying the two ends tightly with a string at equal distance. The bags were then placed in beakers containing 100 mL PBS (pH = 7.4), and were placed in a thermostatically controlled shaking water bath (WSB-18, Daihan Scientific Co. Ltd, Gangwon-do, Korea) at 37 °C and 100 rpm. A volume equal to 1 ml was taken out of the medium and replaced with 1 mL of fresh PBS to retain sink condition and maintain constant volume of the medium. A total of 10 samples were withdrawn at predetermined time intervals (1,2,3,4,5,6,7,8,24 and 48 hrs). The concentration of the extract in the medium was determined spectrophotometerically. The experiment was done in triplicates. The amount of extract released is calculated using the equation (Kim et al., 2016):

$$\text{Release \%}=\;\frac{\text{Amount of extract released fron nanocapsules}}{\text{Amount of extract initially entrapped in nanocapsules}}\;\times \;100$$

#### Thermogravimetric analysis (TGA)

The method was adopted from Xiao et al. (2017) with slight modifications, using a thermogravimetric analyzer (Perkin Elmer Pyris 6). ALEX-M, ALEX-M-PNCs and B-PNCs samples (2–3 mg) were placed on a platinum pan and the measurements were conducted under inert nitrogen at a flow rate of 25 cm^3^/min. The samples were heated within a temperature range of 30 − 1000 °C at a heating rate of 20 °C/min.

#### Fourier transform infrared (FTIR) spectroscopy

FTIR spectra of ALEX-M, ALEX-M-PNCs and B-PNCs were recorded on FTIR spectrometer (Nexus 670 Fourier Transform Infrared). The actual spectra of the nanocapsules were measured from 400 to 4000 cm^−1^ having a resolution of 4 cm^−1^. The measurements were adjusted toward deionized water as the background solvent. Baseline manipulation and data acquisition were gained using Shimadzu IR solution software v1.40. The method used followed the method adopted by Nur and Vasiljevic (2018).

#### Cell viability (MTT) assay

The cell viability assay was conducted in accordance with a previous study by Mao et al. (2018). RAW 264.7 cells were seeded into 96-well plates at a density of 1 × 10^5^ cells/well and were incubated overnight to allow complete attachment to the surface. DMEM supplemented with 10% *v/v* fetal bovine serum and 1% *v/v* penicillin-streptomycin was used as the culture media. All tested samples were dissolved in media containing 0.1% DMSO (*v/v*). Ibuprofen was used as positive control while 0.1% DMSO was used as negative control. Following complete attachment of cells, DMEM was discarded and replaced with fresh new phenol red-free DMEM. RAW 264.7 cells were stimulated with LPS (0.1 µg/mL)/IFNγ (10 U/mL) and treated with various concentrations of the samples followed by incubation for 24 hours. Next, 100 µl of MTT solution (5 mg/ml) was added to each well and the plates were incubated for 4 hours at room temperature. The media was carefully removed and 100 µl DMSO was added to each well to dissolve the formed purple formazan crystals. Absorbance was measured at *λ* = 540 nm (Multiskan Sky Microplate Spectrophotometer, Thermo Fisher Scientific Massachusetts, United States). The following formula was used to determine cell viability percentage (Aisha et al., 2015; Wang et al., 2019):

$$\text{Cell viability \%}=\frac{O.\text{D Treated Cells}-O.\text{D Blank}}{O.\text{D Untreated Cells}-O.\text{D Blank}}*100$$

Where O.D is the absorbance at λ = 540 nm.

The results are presented as mean ± SD of three independent experiments and the median inhibitory concentration (IC_50_) was calculated from the dose response curve using Graphpad Prism (San Diego, CA, USA, version 5).

#### Anti-inflammatory Griess assay

Based on a series of preliminary trials, the best experiment parameters were determined. The minimum toxic dose which demonstrated >85% viability in the cytotoxicity assay was used as the maximum dose in the anti-inflammatory assay. RAW 264.7 macrophages were treated using different concentrations of tested materials dissolved in media containing 0.1% DMSO. Subsequent to incubation for 2 hours, the cells were then stimulated by LPS (0.1 µg/mL)/IFNγ (10 U/mL) and incubated for 24 hours. The supernatant (50 µl) was transferred to new 96-well plate and the levels of NO produced were quantified by adding 50 µl of Griess reagent. The plate was incubated at room temperature for 15 min and the absorbance was measured at *λ* = 540 nm (Multiskan Sky Microplate Spectrophotometer, Thermo Fisher Scientific Massachusetts, United States). The % of NO inhibition was determined with reference to the standard sodium nitrite as per the following equation (Rao et al., 2016; Lee et al., 2019):

$$\text{NO Inhibition }(\%)=[\frac{O.\text{D LPS Treated Cells}-O.\text{D Test}}{O.\text{D LPS Treated Cells}}]*100$$

#### Zebrafish embryo toxicity test (ZFET)

The assessment of acute toxicity in zebrafish embryos was performed according to Fish Embryo Acute Toxicity Test (FET) 236 developed by the organization for Economic Co-operation and Development (Organization for Economic Co-operation and Development (OECD), 2013). The method was adopted from previous toxicity assays with slight modifications (Alafiatayo et al., 2019; Mohamad et al., 2019; Ponrasu et al., 2012; Wibowo et al., 2018). After 4–5 hpf, the embryos were observed under inverted microscope (Nikon-TS100F), and synchronized embryos with normal development were selected for the assay. The embryos were transferred to 96-well plate (Jet Biofil) using a transfer pipette (one embryo/well). At 6 hpf, 200 µl of media containing 0.1% DMSO with or without treatment was added. The well plates were covered to avoid evaporation and incubated at 28 °C ± 1 in a light/dark cycle (10 hours light/14 hours dark). The exposure of embryos to treatment was static, meaning that the sample test solutions were not renewed throughout the experiment to avoid manipulation of the embryos as much as possible (Basnet et al., 2017). The embryos were observed at 24, 48, 72, and 96 hours post fertilization (hpf) using inverted microscope and dead embryos were discarded on daily basis. Several signs of lethality were monitored namely the coagulation of fertilized eggs, lack of somite formation, lack of tail bud detachment from the yolk sac and lack of heartbeat. The survival rate was determined after 96 hours. The 50% lethal concentration (LC_50_) was calculated based on cumulative mortality. The number of hatched embryos was determined after 48 hpf and the percentage of hatchability was calculated using the formula (Shaikh et al., 2019):

$$\text{\% of Hatchability}=\;\frac{\mathrm{No}.\text{ of hatched embryos}}{\text{Initial no}.\text{ of embryos}}$$

The heart beats per minute were recorded at 72 hpf. Heartbeats counting was done using Danioscope software (Noldus, Wageningen, Netherlands).

The data was obtained from three independent replicates. Twelve embryos were used for each group so a total of 240 embryos was used per replicate. A replicate is considered valid when the mortality or malformations of untreated embryos is ≤10% at the end of 96 hours exposure (Wibowo et al., 2018).

#### LPS-induced anti-inflammatory assay in zebrafish embryo

By reference to the toxicity assay, the concentrations used in the subsequent anti-inflammatory study were decided. The method used was adopted from previous studies (Hwang et al., 2016; Kwon et al., 2017; Lee et al., 2013, 2017). Fertilized zebrafish embryos were collected and incubated in E3 media at 28 ± 1 °C. Viable and normally developing embryos were selected at 7–9 hpf and distributed in 12-well plate containing 2 ml of E3 medium (12–15 embryos/well). The embryos were incubated with or without different concentrations of test material for one hour. Inflammation is induced by adding 5 µg/ml LPS for 15–17 hpf. Then, the zebrafish embryos were transferred to fresh medium in 96-well plates and incubated for 1 hour in the dark at 28 ± 1 °C with DAF-FM DA fluorescent dye (5 µM). Subsequently, the embryos were washed with fresh medium and anaesthized using Lidocaine solution (4.2%). Images of the stained dechorionated zebrafish embryos were captured using a fluorescent microscope (FM800TC AmScope) equipped with a Nikon Digital camera (Coolpix W150). Individual zebrafish larvae fluorescence intensity was quantified using ImageJ 1.46r software (Wayne Rasband).

### Ethical consideration

Embryos are nourished from an attached yolk sac until 120 hpf and independent feeding starts from this point forward, therefore no ethical approval is mandatory for the present study since the observation was completed within 96 hpf (<120 hpf). Also, no human subjects were involved.

### Statistical analysis

All experiments and measurements were subjected to independent triplication. The results were analyzed using descriptive statistics and the data were expressed as mean ± standard deviation (SD). D’Agostino and Pearson omnibus normality test was done initially to verify normal distribution of data. Student’s *t* test was used for comparison of two values, while multiple comparisons were carried out by analysis of variance (ANOVA) followed by Tukey’s *post-hoc* test at 95% significance level. The software used for calculation of IC_50_ and LD_50_ was GraphPad Prism Software (Inc. San Diego, CA, version 5.0). Microsoft Excel (version 16.30) was used to calculate EC_50_. Differences between groups were considered significant if *P* < 0.05.

## Results and discussion

### Fabrication of blank polymeric nanocapsules (B-PNCs)

Prior to encapsulation, preliminary experiments were carried out to set the optimal parameters to synthesize B-PNCs with different concentrations of TG, triton X-100 and AlCl_3_ using different homogenization methods. The best results of B-PNCS were obtained with 1% *w/v* TG, 0.1% *w/v* Triton X-100 and 2% AlCl_3_ using magnetic stirrer for homogenization, which showed the smaller particle size diameter and narrower PDI, hence, were selected for encapsulation of ALEX-M. F12 formulation of B-PNCs size (187 nm) was smaller than other preparations as demonstrated in Table 1 and was selected for further studies.

**Table 1.** Particle size and PDI of B-PNCs by DLS technique in different formulations.

Formula	Homogenizer	Overhead stirrer	Magnetic stirrer	% of TG (*w/v*) (%)	% of Triton X-100 (*w/v*) (%)	% of AlCl_3_ (*w/v*) (%)	Mean diameter (nm)	PdI
F1	+	−	–	1	1	2	8409.33 ± 382.51	0.60 ± 0.15
F2	−	+	−	1	1	2	1475.67 ± 67.26	1.00 ± 0.00
F3	−	−	+	1	1	2	846.20 ± 14.77	0.54 ± 0.02
F4	−	−	+	0.5	1	2	804.73 ± 105.8447	0.99 ± 0.02
F5	−	−	+	2	1	2	8720.00 ± 648.77	0.29 ± 0.05
F6	−	−	+	3	1	2	1943.33 ± 1177.17	0.35 ± 0.06
F7	−	−	+	1	1	1	1795.33 ± 543.31	1.00 ± 0.00
F8	−	−	+	1	1	3	1171.33 ± 113.80	0.96 ± 0.07
F9	−	−	+	1	2	2	2483.33 ± 241.83	1.00 ± 0.00
F10	−	−	+	1	0.5	2	806.40 ± 66.33	0.56 ± 0.01
F11	−	−	+	1	0.25	2	533.13 ± 17.05	0.71 ± 0.22
*F12	−	−	+	1	0.1	2	187.46 ± 1.76	0.40 ± 0.01
F13	−	+	−	1	0.1	2	217.63 ± 13.57	0.71 ± 0.06
F14	+	−	−	1	0.1	2	1738.33 ± 275.43	1.00 ± 0.00
F15	−	−	+	1	0.1	1	368.83 ± 4.25	0.41 ± 0.00
F16	−	−	+	0.5	0.1	2	359.63 ± 20.08	0.80 ± 0.06
F17	−	−	+	1.0	0.50	1	433.56 ± 46.9	0.77 ± 0.16

*Represents the optimized formulation.

### Characterization of ALEX-M-PNCs

#### Particle size, PDI and ZP

**Figure 1.**
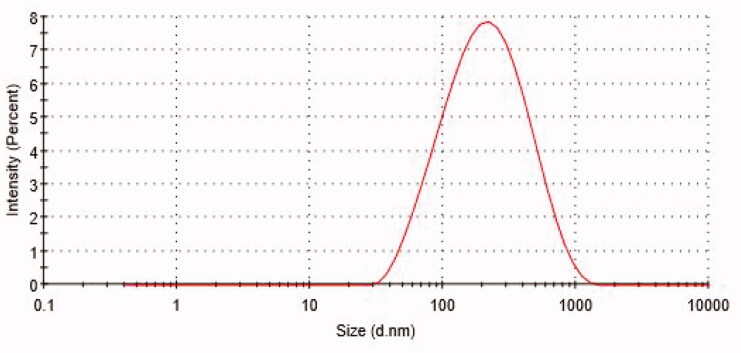
Particle size distribution by intensity of ALEX-M-PNCs measured by DLS technique.

**Figure 2.**
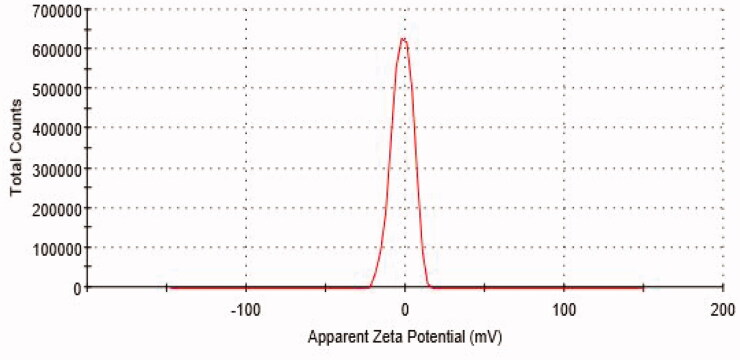
Zeta Potential distribution of ALEX-M-PNCs measured by DLS technique.

The mean particle diameter of ALEX-M-PNCs was equal to 167.13 ± 1.24 nm as shown in Figure 1. The size range is suitable for intravascular injection (Hickey et al., 2015) or topical application (Pal et al., 2011). The PDI value was equal to 0.29 ± 0.01 indicating homogenous particles distribution. A PDI value less than 0.3 suggests narrow size distribution (Kim et al., 2016). The average size and PDI of ALEX-M-PNCs was less than those obtained by other polymeric nanocapsules encapsulating extracts such as *Echinacea purpurea* nanocapsules that demonstrated a size of 218 nm and PDI value of 0.37 (Mao et al., 2018). The ZP value of the synthesized ALEX-M-PNCs was found to be −1.81 ± 0.35 mV as shown in Figure 2. The negative ZP is a good indicator that TG was present at the external surface of the fabricated particles. Although a ZP less than 5 mV indicates fast particles aggregation, this rule is not valid when high molecular weight stabilizers are present (Wu et al., 2011). The low ZP of ALEX-M-PNCs can be due to the neutralization of carboxylic groups of TG with the Al^3+^ trivalent cation of the cross-linker which conjuncts with three carboxylic groups of TG. The same phenomena was observed in turmeric extract-loaded nanocapsules where the ZP was reduced due to the interaction between the carboxylic groups of the wall material with the calcium ions of the cross-linker (Riyajan and Nuim, 2013).

### Morphology

**Figure 3.**
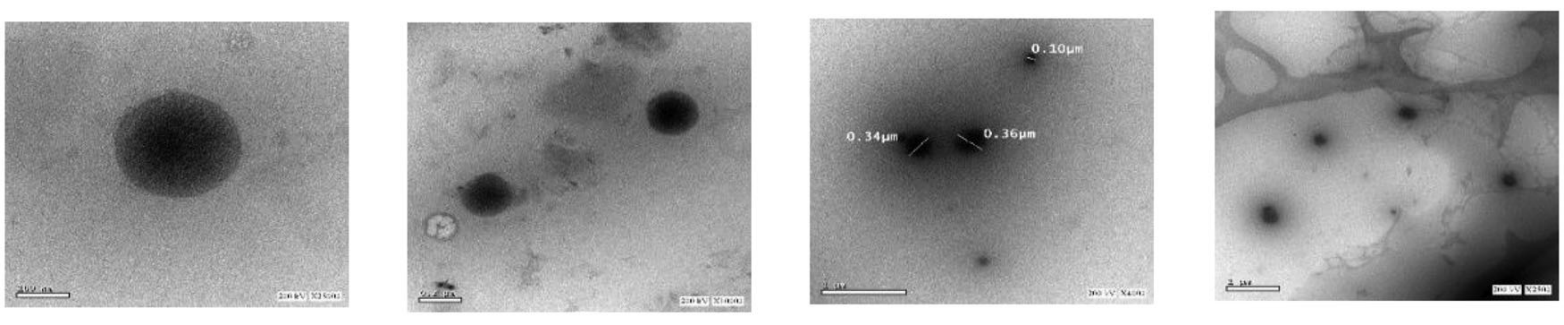
TEM images of ALEX-M-PNCs at different magnifications.

TEM images are shown in Figure 3 confirming the formation of spherical ALEX-M-PNCs with smooth surface.

#### Encapsulation efficiency (EE%) and loading capacity (LC)

ALEX-M was encapsulated into polymeric nanoparticles using w/o/w emulsion method. An EE% of 87.36 ± 1.81% was achieved. An encapsulation efficiency exceeding 70% indicates successful encapsulation of core material (Borges et al., 2018). Based on the amount of encapsulated extract (0.44 mg/ml), LC% was calculated to be 4.4%.

#### In-vitro drug release

**Figure 4.**
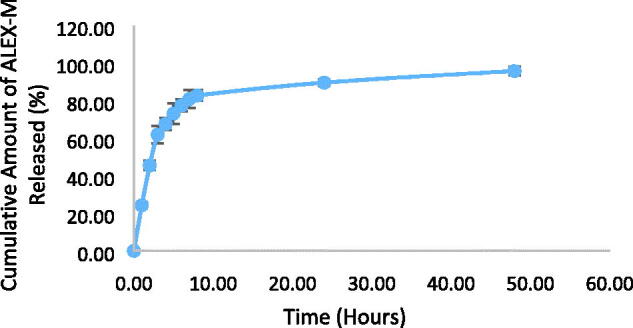
*In-vitro* release of ALEX-M from ALEX-M-PNCs in 48 hours.

The release profile of ALEX-M from the encapsulating nanocapsules over a period of 48 hours is shown in Figure 4. The extract was released in a typical biphasic release pattern characterized by an initial burst and subsequent sustained release. The cumulative release of the extract within the first 8 hours was 82.8 ± 2.8% and additional 13% was released afterwards which resulted in 95.8 ± 2.3% total release of the extract in 48 hours. The two-stage release profile can be related to the design of the polymeric nanocapsules that consist of an inner core and outer shell where the extract can be either adsorbed on the shell surface or dissolved into the core (Stecanella et al., 2013). A similar release pattern was observed with nanoencapsulated apple peel ethanolic extract (Bennet et al., 2014). The sustained release property is desirable because it is associated with reduced doses and side effects.

#### Thermogravimetric analysis (TGA)

**Figure 5.**
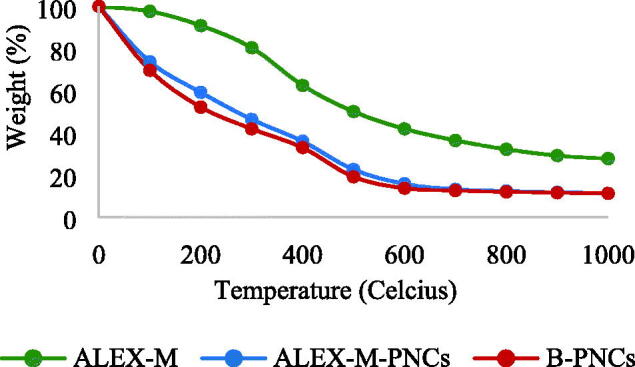
Thermogravimetry diagram of ALEX-M, ALEX-M-PNCs and B-PNCs.

Figure 5 shows the weight loss curves of ALEX-M, ALEX-M-PNCs and B-PNCs obtained by thermogravimetric analysis (TGA). The extract total weight loss was 72.13% at 1000 °C. Three stages can be observed from the curves of the thermal decomposition of ALEX-M-PNCs and B-PNCs. The weight loss in the first stage (30 − 100 °C) of ALEX-M-PNCs and B-PNCs was 26.14 and 31.62%, respectively, and can be attributed to the evaporation of moisture absorbed by the TG polymer hydrophilic in nature (Ghayempour and Montazer, 2016) or the residual of the ethanolic extract adsorbed on the nanocapsules surface. The main weight loss % of ALEX-M-PNCs and B-PNCs occurred in the second stage between 100 and 700 °C and was equal to 59.322 and 55.792% respectively. This can be explained by the rapid release of the core materials and the decomposition of the polymeric wall structure. In the third stage between 700 and 1000 °C, the weight loss in ALEX-M-PNCs and B-PNCs was about 1.8 and 1.28%, respectively caused by evaporation of any residuals. The total weight loss of ALEX-M-PNCs and B-PNCs is 61.122 and 57.072%. The gap is 4.05% which represents the mass fraction of the extract.

The loading capacity was calculated to be 4.98% using TGA curves according to using the formula (Xiao et al., 2017):

$$\frac{61.122\%-\;LC}{1-26.14\%-LC}=\frac{57.072\%}{1-31.62\%}$$

#### Fourier transform infrared (FTIR) spectroscopy

**Figure 6.**
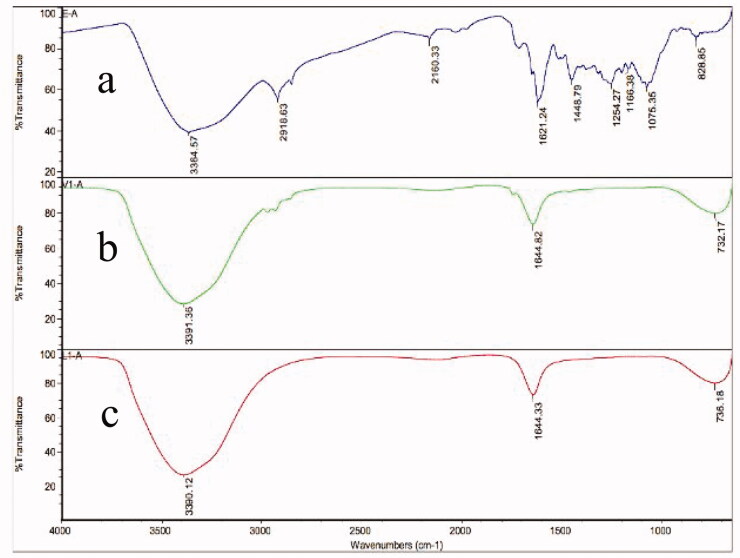
FTIR spectra of (a) ALEX-M, (b) ALEX-M-PNCs and (c) B-PNCs.

The functional groups of ALEX-M, ALEX-M-PNCs and B-PNCs were determined through infrared spectroscopy as demonstrated in Figure 6. The FTIR spectrum of ALEX-M is complex due to inclusion of multiple compounds. The extract showed typical hydrogen-bonded O–H stretch of phenolic compounds at 3364 cm^−1^. Absorption bands occurred at 1448 and 1621 cm^−1^ corresponding to the C = C stretches of aromatic ring. Two peaks appeared at 2918 and 1075 cm^−1^ correspond to C≡C and C–O, respectively. The band at 828 cm^−1^ correspond to the angular deformation outside the plane of aromatic C–H (Pretsch et al., 2009). The FTIR spectra of B-PNCs showed characteristics absorption bands at 3391 cm^−1^ corresponding to stretching vibration of OH groups, and absorption bands at 1644 cm^−1^ representing C=O or COO^−^ that can be due to carboxylic acid or carboxylate anion of d-galacturonic acid present in TG (Hosseini et al., 2016). After encapsulation of the extract, the FTIR spectrum of ALEX-M-PNCs showed that the characteristic absorption peaks 1075, 1166, 1254, 1448, 1621, 2160, and 2918 cm^−1^ of the extract disappeared indicating the extract encapsulation within the nanocapsules. The absorption band at 3364 cm^−1^ was found to overlap with peaks in the broad band range of OH group present in TG wall material. The peaks of ALEX-M-PNCs and B-PNCs were identical which proves that the extract was successfully encapsulated in the polymeric wall materials (Xiao et al., 2017). The absence of chemical bonding between the nanocapsules and the extract as shown in FTIR analysis suggests that the extract was purely physically entrapped without any chemical interaction between the extract and the coating material (Hani et al., 2016; Yamala et al., 2017).

## In-vitro cytotoxicity assay

**Figure 7.**
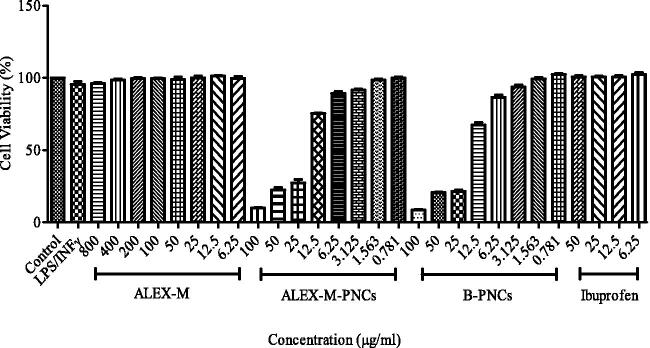
Concentration dependent cytotoxicity effect of LPS/INFγ, ALEX-M, ALEX-M-PNCs, B-PNCs, and Ibuprofen against RAW 264.7 cell line. Cell cytotoxicity was determined using MTT assay. Data are the means ± standard deviation (SD) of three independent experiments.

In order to determine the extract and nanoparticles safest concentrations toward RAW 264.7 macrophages, the samples were subjected to cytotoxicity screening using MTT assay. The results presented in Figure 7 showed that the control group, LPS/IFNγ (0.1 µg ml^−1^/10 IU) and Ibuprofen treated cells showed nearly 100% viability. The results also showed that ALEX-M is nontoxic toward RAW 264.7 cells because the viability percentage was above 95% at all tested concentrations ranging from 800 to 6.25 µg/ml. In contrast, ALEX-M-PNCs and B-PNCs demonstrated cell viability less than 75% at concentrations 100–12.5 µg/ml. However, better safety profile (above 85% cell viability) was detected at concentrations ranging between 6.25 and 0.781 µg/ml of the nanocapsules. The IC_50_ of ALEX-M-PNCs and B-PNCs are 16.60 µg/ml and 13.93 µg/ml, respectively. The similar cytotoxicity profiles of ALEX-M-PNCs and B-PNCs in the tested range of concentrations suggest minor effect of the extract on cytotoxicity. It is unlikely that TG is the reason behind this finding due to its biocompatibility and safe use in food and drug industries (Ghayempour et al., 2015b). It can be presumed that the declination of the cell viability with increasing concentrations of nanocapsules is due to their accumulation in high numbers inside the cells causing increased cellular stress and cell death (Alishah et al., 2017). Another explanation could be that RAW 264.7 macrophages produce several biological mediators including NO as a defense mechanism against stimuli and can undergo NO-dependent cell death (Karabay et al., 2012; Marrassini et al., 2018). The nontoxic concentrations were used in the subsequent anti-inflammatory Griess assay.

## Anti-inflammatory Griess assay

**Figure 8.**
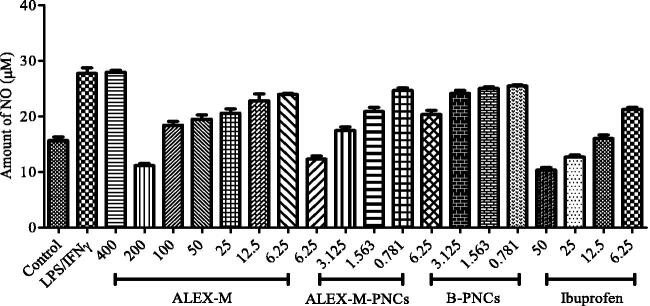
Effect of ALEX-M, ALEX-M-PNCs, and B-PNCS on LPS/IFNγ-induced NO in RAW 264.7 cells. Data are the means ± standard deviation (SD) of three independent experiments, *p* < 0.05. Student’s *t* test was used to compare EC_50_ values at *p* < 0.05.

The inflammatory mediator NO^·^ reacts with oxygen to produce stable nitrate (NO_3_−) and nitrite (NO_2_^·^) that can be quantified using Griess reagent (Islam et al., 2016). As shown in Figure 8, LPS/IFNγ stimulation demonstrated significant increase (*p* < 0.05) in NO production (27.708 µM) compared to control group (15.649 µM). The extract at a concentration of 400 µg/ml showed an undesirable stimulating effect on NO production, while it showed significant reduction in NO production ranging from 58.124% to 13.090% at concentrations between 200–6.25 µg/ml compared to LPS/IFNγ-stimulated group (Figure 8). ALEX-M exhibited NO inhibitory activity with an EC_50_ value of 164.790 ± 6.318 µg/ml, compared to the standard Ibuprofen with an EC_50_ value of 30.91 ± 5.878 µg/ml. The finding signifies the anti-inflammatory activity of the extract, which can be attributed to the presence of phenolic compounds as recommended by previous studies (Mennen et al., 2004; Okoli and Akah, 2004). In a previous study, a number of phorbol esters isolated from *A. malaccensis* seeds obtained from Taiwan the demonstrated potent anti-inflammatory activity (Wagh et al., 2017). Similarly, sesquiterpenes derivatives isolated from agarwood chips of *A. malaccensis* tree obtained from an industrial plantation in Laos, showed ability to suppress inflammation (Thanh et al., 2021). The result of the present study is compatible with the anti-inflammatory activity of *A. malaccensis* leaf extract demonstrated through its ability to inhibit lipoxygenase enzyme (Eissa et al., 2020).

The results exhibited in Figure 8 showed that RAW264.7 cells pretreated with ALEX-M-PNCs at concentrations ranging from 6.25–0.781 µg/ml demonstrated dose dependent inhibition in NO production of 53.92–10.56%. The EC_50_ value of ALEX-M-PNCs is 5.50 ± 0.31 µg/ml which is lower than EC_50_ of Ibuprofen (30.91 ± 5.878 µg/ml). It is obvious that nano-encapsulation potentiated the pharmacological anti-inflammatory effect of ALEX-M. To clarify, ALEX-M-PNCs at a concentration of 6.25 µg/ml inhibited NO production by 53.92 ± 3.65% which is not statistically different (*p* < 0.05) from ALEX-M at 200 µg/ml that is equal to 58.12 ± 1.50%. Similar results were attained in a previous study as the anti-inflammatory activity of rosiglitazone was enhanced due to increased cellular uptake of the nano-encapsulated rosiglitazone, delivering higher amount of the treatment to the site of action (Giacalone et al., 2018). It was also found that the amount of NO was slightly reduced by increasing concentrations of B-PNCs, which can be attributed to the composition of the nanocapsules. The significant difference (*p* < 0.05) between ALEX-PNCs and B-PNCs in NO reduction supports the anti-inflammatory effect of the extract.

**Figure 9.**
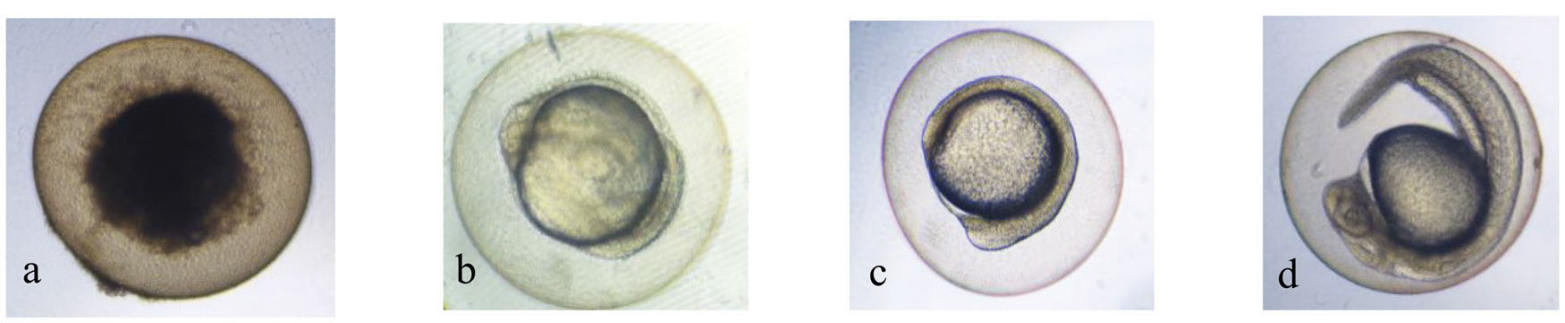
Signs of lethality in zebrafish embryos: (a) coagulation (b) non-detachment of tail (c) lack of somite formation (d) normal embryo showing no signs of toxicity at 24 hpf. Images were captured under magnification of 40×.

## Zebrafish embryos toxicity assay

### Survival rate

The toxicity of ALEX-M, ALEX-M-PNCs and B-PNCs was studied in zebrafish embryos with test time of 96 hours in accordance with OECD 236 protocol and the signs of lethality are demonstrated in Figure 9.

**Figure 10.**
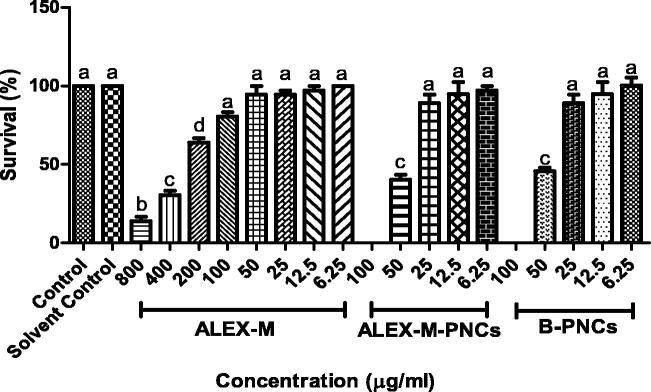
Dose response to mortality of zebrafish embryos at 96 hpf, exposed to ALEX-M (6.25–800 µg/ml) and ALEX-M-PNCs and B-PNCs at concentrations (6.25–100 µg/ml) (*n* = 12 embryos/group) of three independent replicates. Statistical analysis was performed using one-way ANOVA followed by Tukey’s *post-hoc* test. Different letters (a–d) indicate significant difference at *p* < 0.05.

The cumulative survival percentage of zebrafish embryos treated with samples for 96 hpf is presented in Figure 10. It was observed that the toxicity effects of ALEX-M were dose-dependent at concentrations from 800 to 6.25 µg/ml. The control group and the extract at lower concentrations (≤12.5 µg/ml) demonstrated 100% viability in zebrafish embryos. However, the survival rate decreases significantly with higher concentrations of the extract (≥200 µg/ml), indicating the sensitivity of zebrafish embryos to high concentrations of ALEX-M at early developmental stages. The LD_50_ value of ALEX-M was found to be 207.0 µg/ml and therefore is considered safe according to OECD guidelines (Shaikh et al., 2019). This finding was supported by a previous study where ALEX-M toxicity was evaluated in Sprague-Dawley Rats and demonstrate no signs of toxicity at a dose up to 2000 mg/kg (Liyana et al., 2018). According to Zhang et al., (2003) and Basnet et al., (2017), there is a correlation between zebrafish and rodents toxicity.

As for ALEX-M-PNCs and B-PNCs-treated groups, similar toxicity profile of ALEX-M was observed and the survival rate was dose-dependent at a concentration range of 50–6.25 µg/ml as shown in Figure 10. The viability of zebrafish embryos was found to be ≥95% at 6.25 µg/ml of ALEX-M-PNCs and B-PNCs, and was declined gradually with increasing concentrations. All the embryos died after exposure to 100 µg/ml of ALEX-M-PNCs and B-PNCs within 24 hpf. The LD_50_ values of ALEX-M-PNCs and B-PNCs do not differ significantly and were calculated as 49.63 and 49.60 µg/ml, respectively. This suggests negligible effect of the extract on the nanoparticles toxicity. In general, the exposure of zebrafish embryos to nanoparticles increases their mortality (Chakraborty et al., 2016). Cellular apoptosis in zebrafish embryos was detected upon treatment with chitosan nanoparticles (Hu et al., 2011), therefore it is possible that TG nanocapsules have employed similar behavior. Although natural, biocompatible and biodegradable polymers are safe, they showed potential toxicity when used in synthesizing nanocarriers.

### Hatching rate

Hatching of embryos occurs within 48-72 hpf (Brundo and Salvaggio, 2018). A zebrafish embryo is considered as hatched when a complete rupture of the chorion occurs and the entire body of larvae from head to tail is out (Alafiatayo et al., 2019; Shaikh et al., 2019). Hatching is a direct indication of the successful developmental processes of zebrafish embryos (Shanmugapriya et al., 2019), and is considered an important parameter to understand the toxicity of chemicals and nanomaterials (Chakraborty et al., 2016). Moreover, the complete inhibition of hatching can lead to death of embryo inside the chorion (Ong et al., 2014). According to Organization for Economic Co-operation and Development (OECD) (2013), if the survival and hatching rates are respectively 90% and 80%, the sample is assumed to be nontoxic (Amora et al., 2018). The hatching rate was expressed as the number of embryos hatched after 72 hpf, as compared to control (Ponrasu et al., 2012).

**Figure 11.**
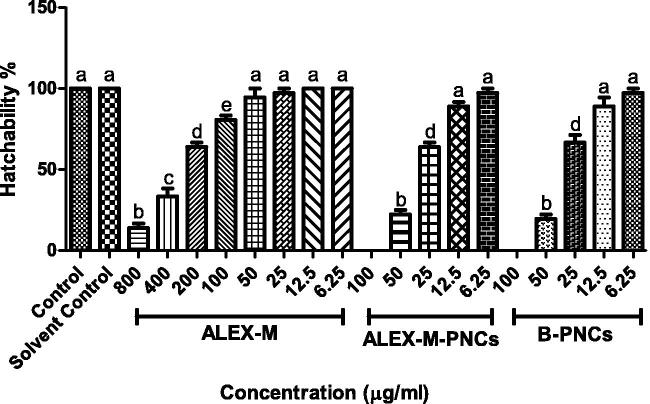
Effect of different concentrations of the tested samples on hatchability percentage in zebrafish embryo. Values are expressed as mean ± SD (*n* = 12 embryos/group) of three independent replicates. Statistical analysis was performed using one-way ANOVA followed by Tukey’s *post-hoc* test. Different letters (a–e) indicates significant difference at *p* < 0.05.

As seen in Figure 11, ALEX-M-treated embryos demonstrated significant reduction in the hatchability at concentrations ≥100 µg/ml, while no significant difference in the hatching rate was observed when compared to control at concentrations ranging between 50–6.25 µg/ml.

The highest hatching rate of the embryos exposed to ALEX-M-PNCs and B-PNCs was obtained at 12.5 and 6.25 μg/ml, and the rate dramatically decreased at concentrations between 100-25 μg/ml. The findings are consistent with a previous study where significant reduction in hatching rate upon exposure to multiple nanoparticles was observed (Ong et al., 2014). Also, chitosan nanoparticles demonstrated dose-dependent decline in hatching rate with increasing concentrations of nanocapsules from 5 to 30 µg/ml (Nikapitiya et al., 2018). According to Ong et al., (2014), several nanoparticles were reported to affect the early development of zebrafish which can delay the maturation of the hatching gland and subsequently inhibit the hatching enzyme resulting in a longer incubation within the chorion (Ong et al., 2014). Chakraborty et al. (2016) suggested that interaction occurs between nanoparticles and hatching enzymes causing inhibition of hatching and death of embryos. Since ALEX-M-PNCs and B-PNCs demonstrated similar effect on the hatchability of embryos, then it can be concluded that extract has no or minor effect on hatchability. Accordingly, the reduced hatching rate in embryos exposed to nanocapsules can be attributed to TG which increases the viscosity of the medium causing high resistance against dispersion according to (Ghayempour et al., 2016b).

### Heart rate

**Figure 12.**
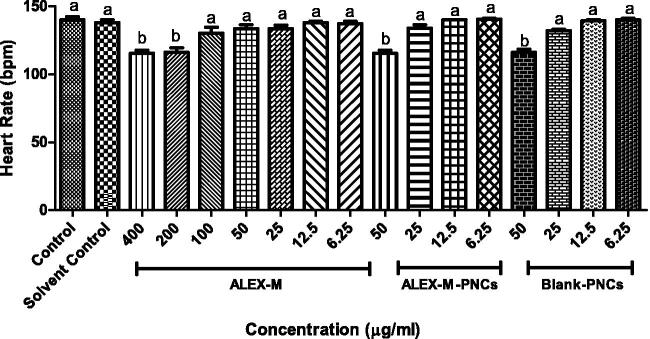
Effect of different concentrations of the tested samples on the heart rate in zebrafish embryo. Values are expressed as mean ± SD (*n* = 3 embryos/group) of three independent replicates. Statistical analysis was performed using one-way ANOVA followed by Tukey’s *post-hoc* test. Different letters (a–b) indicates significant difference at *p* < 0.05.

The heart is the first internal organ formed in zebrafish embryos and is usually developed within 24–48 hpf (Fahmi et al., 2017; Luca et al., 2015). The heart rate is considered an important parameter in determining several physiological effects (Shanmugapriya et al., 2019) and a significant endpoint of sublethal toxicity (Shaikh et al., 2019). The normal heart rate in embryonic zebrafish is 120–180 beats per minute (Luca et al., 2015). Figure 12 presents the effect of the tested samples on the heart rate.

Control embryos show normal heartbeat at 96 hpf (140 bpm). Significant decrease in the heart rate was observed at concentrations ≥200 µg/ml concentration of ALEX-M as compared to control group, while no significant reduction in heart rate occurred with concentrations ≤100 µg/ml (Figure 12). Therefore, it can be anticipated that the heart rate of zebrafish larvae might be a potential target for toxicity by high concentrations of ALEX-M. Several previous studies have shown the effect of plant extracts on the heart rate. For instance, the aqueous extract of *Ficus glomerata* reduces heart rate in zebrafish embryos in a dose-dependent manner (Fahmi et al., 2017).

**Figure 13.**
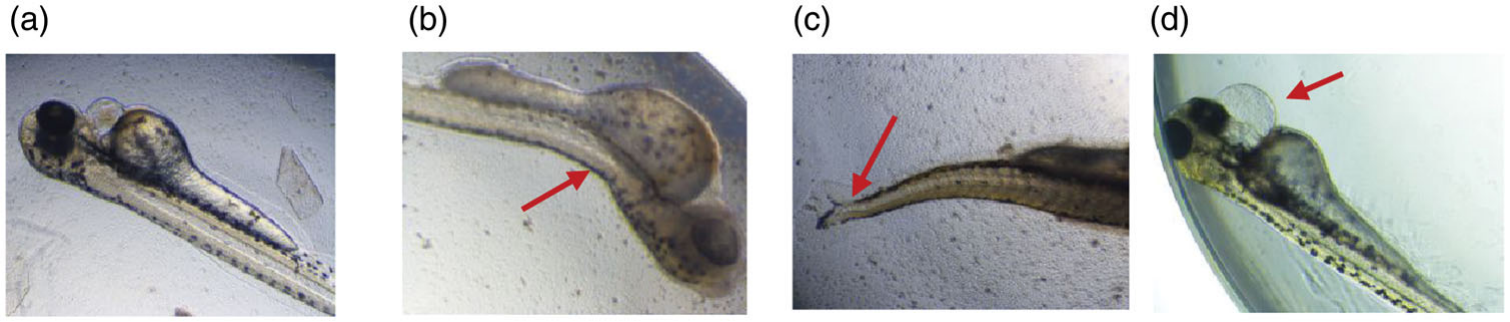
Malformations in zebrafish larva: (a) normal larva at 72 hpf, (b) spinal curvature, (c) scoliosis, (d) pericardial edema. Images were captured under magnification of 40×.

As for the ALEX-M-PNCs and B-PNCs, a significant reduction in the heart rate at a dose of 50 µg/ml was observed. There was no statistically significant difference between ALEX-M-PNCs and B-PNCs, which confirmed that the reduced heart rate is not due to the encapsulated extract. The abnormal heart rate and impaired blood circulation may be due to weakened cardiac functions caused by immature heart and consequent delay of body growth (Majewski et al., 2017; Shaikh et al., 2019).

### Body malformations

Figure 13 demonstrate the malformations in zebrafish larva that were detected in the present study including spinal curvature, scoliosis and pericardial edema. No body malformations occurred with the ALEX-M-treated embryos at all tested concentrations throughout 96 hpf. On the other hand, morphological abnormalities occurred in some zebrafish embryos treated with nanocapsules at a concentration ≥50 µg/ml. After ruling out the effect of the extract on the development of body malformations, these manifestations can be correlated with the coating materials used in nanoparticles. It can also be attributed to the accumulation of nanocapsules in body tissues which can lead to serious body deformities (Ong et al., 2014).

**Figure 14.**
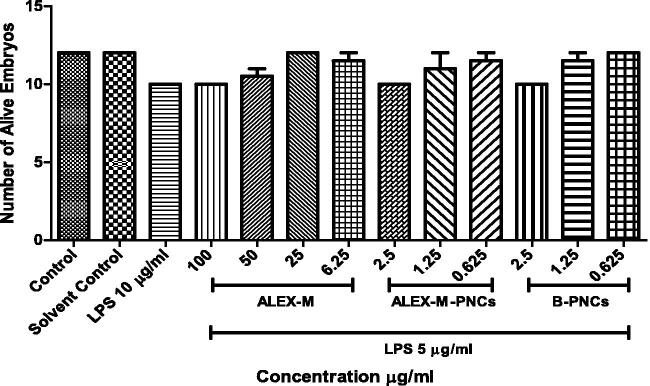
Dose response of embryonic zebrafish mortality at 96 hpf, exposed to ALEX-M (6.25–100 µg/ml) and ALEX-M-PNCs and B-PNCs at concentrations (0.625–2.5 µg/ml), (*n* = 12 embryos/group) in three replicates. Statistical analysis was performed using one-way ANOVA followed by Tukey’s *post-hoc* test. No significant difference occurred between all groups at *p* < 0.05.

### Anti-inflammatory assay in zebrafish embryos

Prior to evaluation of the NO inhibitory effect of the samples, the survival rate of zebrafish embryos was first examined in the presence of LPS (5 µg/ml) to determine safe concentrations of the samples as shown in Figure 14. Embryos were treated concomitantly with LPS 5 µg/ml and ALEX-M at concentrations of 100, 50, 25 and 6.25 µg/ml, ALEX-M-PNCs at concentrations of 2.5, 1.25 and 0.625 µg/ml corresponding to the concentrations of 2.165, 1.082, and 0.541 µg/ml of the encapsulated extract.

**Figure 15.**
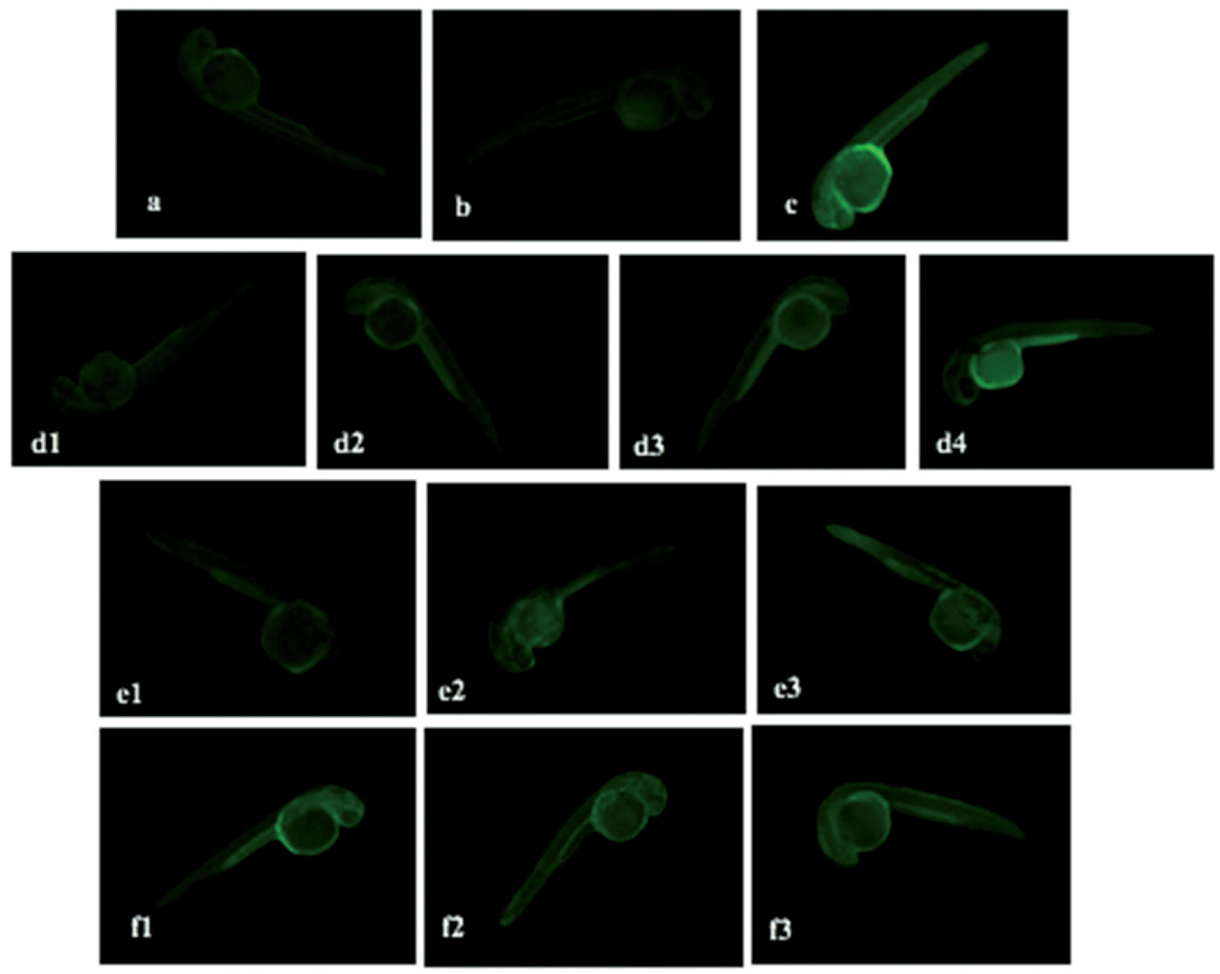
Fluorescence micrographs of LPS-stimulated NO generation in zebrafish embryos showing the effect of ALEX-M, ALEX-M-PNCs and B-PNCs on LPS-induced NO production. The images were visualized using 40× magnification power. (a) control, (b) ALEX-M 100 µg/ml, (c) LPS 5 µg/ml, (d1) ALEX-M 100 µg/ml + LPS 5 µg/ml, (d2) ALEX-M 50 µg/ml + LPS 5 µg/ml, (d3) ALEX-M 25 µg/ml + LPS 5 µg/ml, (d4) ALEX-M 6.25 µg/ml + LPS 5 µg/ml, (e1) ALEX_M_PNCs 2.5 µg/ml + LPS 5 µg/ml, (e2) ALEX_M_PNCs 1.25 µg/ml + LPS 5 µg/ml, (e3) ALEX_M_PNCs 0.625 µg/ml + LPS 5 µg/ml, (f1) B-PNCs 2.5 µg/ml + LPS 5 µg/ml, (f2) B-PNCs 1.25 µg/ml + LPS 5 µg/ml, (f3) B-PNCs 0.625 µg/ml + LPS 5 µg/ml.

**Figure 16.**
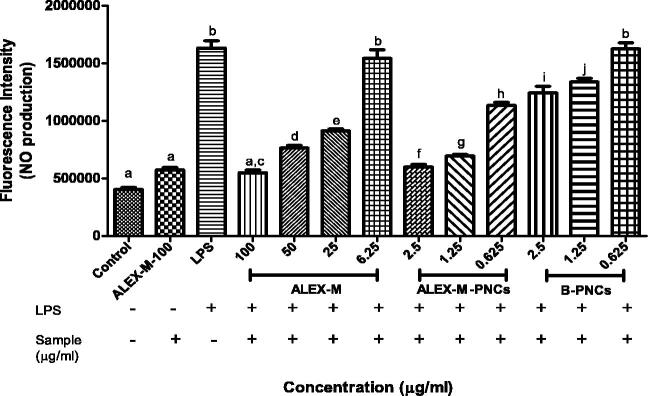
Quantitative analysis of fluorescence intensity of zebrafish embryos after staining with DAF-FM-DA using imageJ program. Data are represented as means ± SD from three independent experiments (*n* = 9 embryos/group). Statistical analysis was performed using one-way ANOVA followed by Tukey’s *post-hoc* test. Values with different letters (a–j) are significantly different in comparison to negative and positive control at *p* < 0.05.

Figure 15 demonstrates the fluorescence intensity in LPS-stimulated zebrafish embryos treated with different samples after staining with DAF-FM-DA, while Figure 16 presents the quantitative analysis of fluorescence intensity of zebrafish embryos using imageJ program. The negative control group and ALEX-M only treated group at 100 µg/ml generated a dark image with no significant difference in NO levels, thus indicating that ALEX-M alone did not significantly increase the basal NO levels. Meanwhile, LPS treatment significantly increased NO production to 406.057% when compared to negative control and generated highly fluorescent image. In zebrafish embryos treated with ALEX-M prior to LPS, a reduction in the fluorescence intensity occurred indicating reduction in LPS-stimulated NO production in a concentration-dependent manner. Treatment with ALEX-M at 100, 50, 25 and 6.25 µg/ml significantly reduced the level of NO to 136.623, 191.528, 228.698 and 384.064%, respectively, in LPS-treated zebrafish. As for ALEX-M-PNCs-treated group, the level of NO was reduced to 259.399, 173.999, and 150.133%, at concentrations of 0.625, 1.25 and 2.5 μg/ml, respectively, in LPS-stimulated embryos. The reduction in NO level by ALEX-M-PNCs at 2.5 µg/ml is comparable to that obtained by 100 µg/ml ALEX-M (136.623%) showing no statistically significant difference between the two groups. It is obvious that the anti-inflammatory effect of ALEX-M was potentiated by nano-encapsulation. This can be attributed to higher surface areas arising from nano-sized particles, which results in increased cellular uptake and absorption (Kim et al., 2016). Thus, nanocapsules has better penetration through the chorion surrounding the zebrafish embryos. It can be noted that B-PNCs at 1.25 and 2.5 µg/ml demonstrated significant reduction in NO level compared to LPS treated embryos. These findings indicate that there might be synergistic anti-inflammatory activity between the extract and the wall material of the nanocapsules (TG).

## Conclusion

In this study, nano-encapsulation has been used to improve the efficacy of herbal extracts. ALEX-M was encapsulated in TG and the fabricated nanocapsules were characterized and evaluated for their morphology, particle size, PDI, ZP, EE, LC, TGA, FTIR and extract release. The characterization of the ALEX_M_PNCs demonstrated the extract encapsulation with high efficiency in spherical shape with smooth surface and in nano size range. The in-vitro anti-inflammatory assay revealed suppression of NO production in LPS-stimulated RAW 264.7 cells by the free and nano-encapsulated extract. The toxicity and the anti-inflammatory activity of the free and nano-encapsulated extract were evaluated in zebrafish embryos and demonstrated dose-dependent toxicity and anti-inflammatory activity. The results verified the potentiation of the anti-inflammatory activity of the extract via nano-encapsulation. The findings gained from this study support the use of ALEX-M as a potential agent for the treatment of inflammatory disorders. Nevertheless, further studies are needed to elucidate the mechanisms underlying the anti-inflammatory effects of the extract, which will be beneficial to develop future natural anti-inflammatory agents.
